# Determinants of Acute Kidney Injury in Children With Nephrotic Syndrome: A Prospective Observational Study

**DOI:** 10.7759/cureus.76878

**Published:** 2025-01-03

**Authors:** Nimisha Mohanty, Anil Kumar Goel, Manas R Sahoo, Seema Shah, Ipsa Mohapatra

**Affiliations:** 1 Pediatrics, All India Institute of Medical Sciences, Raipur, Raipur, IND; 2 Biochemistry, All India Institute of Medical Sciences, Raipur, Raipur, IND; 3 Community Medicine, Kalinga Institute of Medical Sciences, Bhubaneswar, IND

**Keywords:** aki, children, nephrotic syndrome, sickle cell disease, underhydration

## Abstract

Background

Nephrotic syndrome (NS) is a common renal ailment among children, typically manifesting as a relapsing-remitting pattern. Most of the cases are managed on an outpatient basis, but a subset of patients experience complications, e.g., acute kidney injury (AKI). Although historically more prevalent in secondary NS, AKI is now occurring increasingly in children with idiopathic NS. However, the literature on AKI in this population consists of case reports and retrospective studies, particularly from India, so the study was planned to identify various risk factors that precipitate AKI in a child with NS. The secondary objective was to assess the hydration status of children having NS and its association with the development of AKI.

Materials and methods

This longitudinal study was conducted in the Department of Pediatrics at the All India Institute of Medical Sciences, Raipur, Chhattisgarh, from October 2021 to April 2023. Children of both genders and age groups between three months and 15 years, satisfying the International Society for Pediatric Neurosurgery 2021 guideline for the diagnosis of NS, were included in the study. Children having chronic kidney disease were excluded. Using the non-probability convenient sampling technique, 57 patients with NS were enrolled in the study. The patients without AKI were evaluated daily for the development of AKI using the Kidney Disease Improving Global Outcomes 2012 guideline until day 14 or discharge and followed up for six months. Records of those children who were admitted with AKI were reviewed for possible risk factors of AKI. Data was analyzed on Epi Info software enUS version 7.3.2 (Centers for Disease Control and Prevention, Atlanta, GA, USA). Categorical data were expressed as a percentage and/or 95% confidence interval (CI) of the estimate and compared using Chi-square or Fisher's exact test. A p-value ≤0.05 was considered statistically significant. The odds ratio (OR) for risk factors for AKI was determined using logistic regression.

Results

The mean age of the study subjects at the onset of the disease was 5.34 ± 3.66 years. The common presentations were edema (94.74%) and oliguria (80.7%). The majority (89.2%) showed a response to steroid therapy. About 56.14% of children developed AKI, and stages 2 and 3 AKI were more common, 37.5% each. About 53.12% and 46.88% of children developed pre-renal AKI and intrinsic AKI, respectively; 45.61% had hypertension at admission, with the majority having stage 1 hypertension (38.46%). Only six (10.5%) children had sickle cell trait, and all developed AKI during follow-up. Forty-two (73.68%) children had nephrotoxic drug exposure, with the most common drug being enalapril, followed by nephrotoxic antibiotics. Out of 10 children with AKI who underwent renal biopsy, focal segmental glomerulosclerosis was the most common entity (60%). The notable parameters that were found to have statistical significance for AKI were low eGFR at admission, hypertension, nephrotoxic drug exposure, inadequate water intake, fractional excretion of sodium (FeNa), and urine potassium index as markers of renal hypoperfusion, infections, steroid-resistant nephrotic syndrome, and significant glomerular lesions.

Conclusion

The present study demonstrates an association between traditional risk factors and the causation of AKI. However, high urine osmolality, raised urine K+ index, and FeNa suggestive of raised aldosterone levels had a significant association with AKI.

## Introduction

Nephrotic syndrome (NS) is a common renal disease in children, characterized by heavy proteinuria, hypoalbuminemia, and edema, typically manifesting as a relapsing-remitting pattern [1]. While the idiopathic variant (INS) represents the common variety, secondary causes such as systemic lupus erythematosus, Henoch-Schoenlein purpura, malignancies (lymphoma and leukemia), and infections (hepatitis, HIV, and malaria) [2] do present as NS. The global incidence of INS exhibits variability, with a notably higher prevalence observed among individuals of South Asian descent, 1-18 cases per 100,000 population [3].

Although many cases are managed on an outpatient basis, a subset of patients experience complications, including severe infections, hypovolemia, venous thromboembolism, and acute kidney injury (AKI). Notably, the incidence of AKI, once considered rare in INS, appears to be on the rise [4]. The epidemiology and outcomes of AKI remain to be elucidated in a larger perspective [5].

AKI in NS is attributed to various etiological factors, which might represent reversible renal vasoconstriction during relapse caused by hypovolemia, interstitial nephritis, nephrotoxic drugs, infections, acute tubular necrosis, or renal vein thrombosis [1].

Patients with sickle cell disease (SCD) exhibit chronic hemolysis fostering endothelial nitric oxide dysfunction, exacerbated by endothelin-1 overexpression, thus compromising renal compensatory mechanisms and heightening susceptibility to AKI. Underhydration occurs when an individual has low water intake without signs of total body water (TBW) deficit, thirst, or elevated plasma osmolality yet triggers water homeostatic mechanisms [6].

Although not accompanied by a TBW deficit, underhydration may contribute to AKI in NS children with low water intake and increase the risk of renal injury via renal stone formation or UTI acquisition [7].

Although historically more prevalent in secondary NS, AKI is now increasingly recognized in children with INS. However, the literature on AKI in this population predominantly consists of case reports and retrospective studies, particularly from India.

Our study was carried out in Central India, the sickle cell belt of India. The data regarding renal injury in children with SCD is very sparse in the literature in this part of the subcontinent. Given the documented prevalence of SCD in central India and renal involvement (10%), further insight into renal complications in patients with NS and SCD was a felt need. Dehydration is a known risk factor for AKI. However, the potential impact of reduced fluid intake compared to daily normal requirements, leading to underhydration, and its impact on renal function is yet to be reported. The present study was planned to find out whether underhydration plays a role in the causation of AKI in children with NS.

The primary objective was to study various risk factors that precipitate AKI in children with NS. The secondary objective was to assess the hydration status of children having NS and its association with the development of AKI.

This study has been presented as a poster in ISPNCON 2023, held in Kolkata, and as an oral paper presentation in CGPEDICON-2023 at Bhilai.

## Materials and methods

Study setting

Using a longitudinal study design, the study was conducted from 16 October 2021 to 23 April 2023 in the Department of Pediatrics at the All India Institute of Medical Sciences (AIIMS), Raipur.

Study participants

Children aged three months to 15 years who met the diagnostic criteria for NS as outlined by the Indian Society of Pediatric Nephrology 2021 guideline [8] were included in the study. However, children with chronic kidney disease were excluded.

The sample size was calculated based on the proportion of risk factors responsible for AKI (estimating the difference between two proportions based on risk difference), taking into consideration the study by Kushwah et al. [4]:

$n = Z^2 \left(\frac{1 - \alpha}{2}\right) \cdot \frac{P_1(1 - P_1) + P_2(1 - P_2)}{d^2}$

where P1 is the proportion in the first group, P2 is the proportion in the second group, d2 is the population risk difference, and 1-α is the desired confidence level.

**Figure 1:**
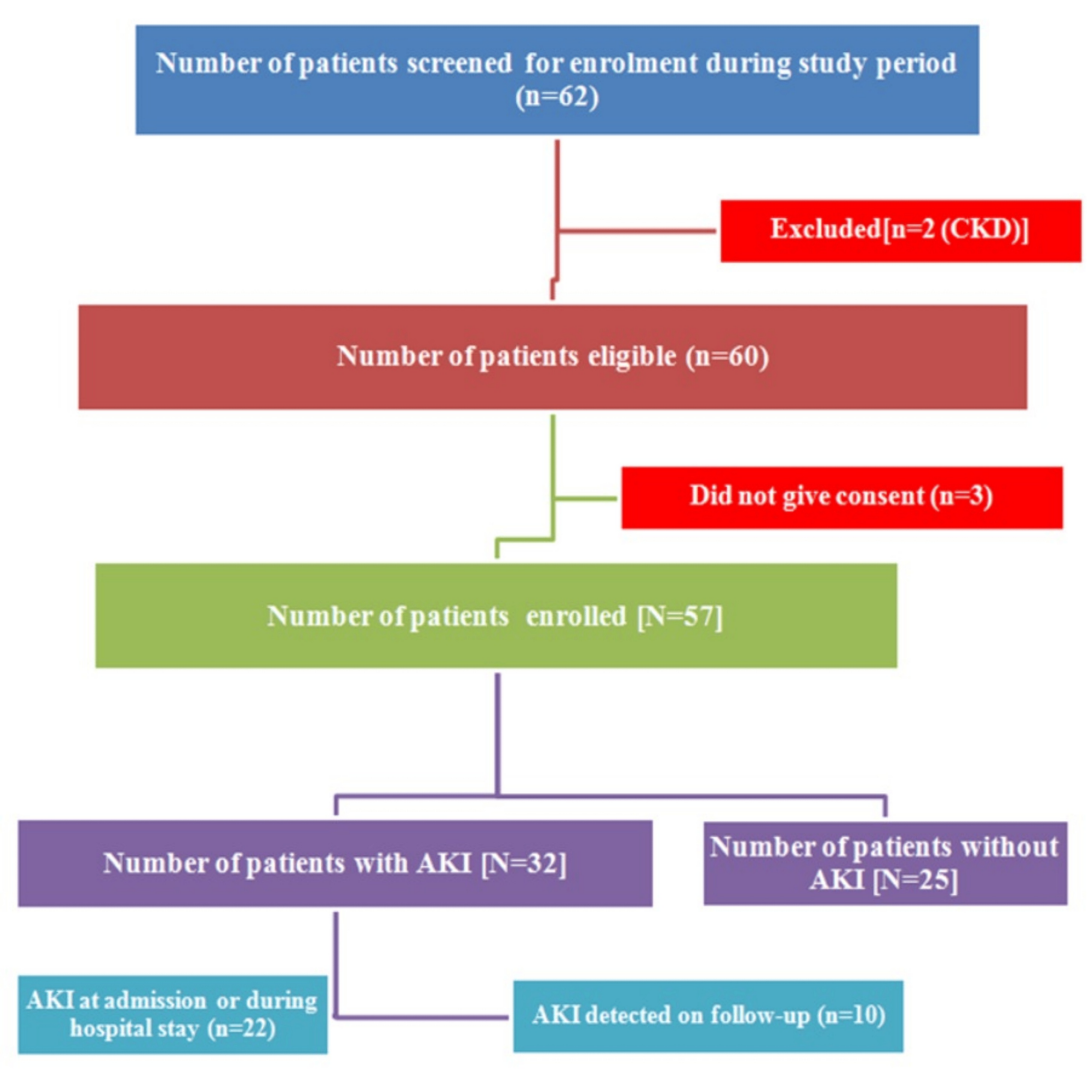
Sample size (N=57) CKD: chronic kidney disease, AKI: acute kidney injury

The sample size was calculated as 56, with a confidence level of 95% and a precision level of 10%. Using the non-probability convenient sampling technique, 57 patients who were admitted to the Department of Pediatrics at AIIMS, Raipur, and satisfied the inclusion criteria were enrolled in the study after obtaining written informed consent from parents and assent, wherever appropriate (Figure 1).

Study procedure

The children were categorized into groups of those with AKI and those with no AKI after screening. The study was approved by the Institutional Ethics Committee of the All India Institute of Medical Sciences, Raipur (approval number: AIIMSRPR/IEC/2021/942, approval date: October 1, 2021).

All the relevant data, including the demographic, clinical history, examination, and supported investigation, were recorded in a structured format. Daily evaluation of patients for AKI using the Kidney Disease Improving Global Outcomes (KDIGO) 2012 guideline [9] until day 14 or discharge, along with clinical and biochemical information to determine associated risk factors. The patients who were admitted with AKI or developed AKI during the hospital stay were treated as per protocol, and detailed history was taken for exposure to risk factors and recorded. Ultrasound of the kidney, including ureter and bladder (USG KUB), was done at admission for the patients suspected to have renal etiology of AKI, and a biopsy was done as per indication. The children who did not develop AKI during the hospital admission were followed up for six months after enrollment. Five follow-ups were done: the first after one week post-discharge, the second after the induction phase, the third after the maintenance phase, and then two more times at intervals of one and a half months to determine if the risk factors persisted and whether they developed AKI.

Most follow-ups (n=7/10) were done physically, and those who could not come due to COVID-19 restrictions had virtual follow-ups done (telephonically).

AKI's definition and staging were done per the KDIGO 2012 guideline [9]. All the genotypic variants of SCD were planned to be included in the study after screening with the sickling test and categorization by high-performance liquid chromatography. The cut-off of water intake per day was taken from median total water intake values in the National Health and Nutrition Examination Survey III survey database, and all the parents were asked about the water intake by the children using the 24-hour recall method [10].

Data collection and statistical analysis

All data were coded and entered into a Microsoft Excel spreadsheet (Microsoft Corporation, Redmond, WA, USA), and data analysis was done on Epi Info software enUS version 7.3.2 (Centers for Disease Control and Prevention, Atlanta, GA, USA). Categorical data were expressed as a percentage and/or 95% confidence interval (CI) of the estimate and compared using Chi-square or Fisher's exact test. Continuous data were expressed as median (interquartile range (IQR)) or mean ± standard deviation (SD) and compared using parametric or nonparametric tests, as appropriate; a p-value of p≤0.05 was considered statistically significant. The Fisher exact test was applied when >20% of cells had frequencies <5. The odds ratio (OR) for risk factors for AKI was determined using logistic regression analysis.

## Results

Of the 57 children admitted, the mean age at onset of AKI was 5.10 (SD ± 3.84) years. Their mean age at admission was 6.93 years (SD ± 3.75); the median age was six years (IQR 4-10 years); 56.14% developed AKI during the study period. Ascites/pleural effusion was present in 97% and 60% of cases in those with AKI and without AKI, respectively, and this difference was statistically significant (p=0.002). Similarly, the variables that showed a difference in both groups and were also found to have a statistically significant association were the episode of infection (p=0.01), hypertension at admission (p=0.03), steroid-sensitive nephrotic syndrome (SSNS) (0.006), frequently relapsing nephrotic syndrome (IFRNS) (0.008), steroid-resistant nephrotic syndrome (SRNS) (0.006), raised procalcitonin (p=0.02), high urine osmolality (p=0.004), and intake of nephrotoxic drugs (p=0.02) (Table 1).

**Table 1:** Comparative analysis of factors of AKI versus non-AKI (n=57) * Chi-square test, ** T-test, # Fisher's exact test, degree of freedom for Chi-square = 1 DVT: deep vein thrombosis, SSNS: steroid-sensitive nephrotic syndrome, SDNS: steroid-dependent nephrotic syndrome, FRNS: frequently relapsing nephrotic syndrome, IFRNS: infrequently relapsing nephrotic syndrome, SRNS: steroid-resistant nephrotic syndrome, CRP: C-reactive protein, eGFR: estimated glomerular filtration rate, AKI: acute kidney injury

Parameters	AKI		p-value	Test of significance value
	Present (n=32) (%)	Absent (n=25) (%)		
Gender				
Female (n=23)	15 (46.88%)	8 (32.00%)	0.38*	0.75*
Male (n=34)	17 (53.12%)	17 (68.00%)		
Age at admission				
Mean (years)	6.12 ± 3.90	7.96 ± 3.32	0.07**	Mean difference 1.84; t=1.88; -12 to 3.791 (95%CI)
Age group (in years) at disease admission				
≤1 year (n=3)	3 (9.38%)	0 (0.00%)	0.19^#^	0.25^#^
1-10 years (n=40)	22 (68.74%)	18 (72.00%)	0.79*	0.07^* ^
≥10 years (n=14)	7 (21.88%)	7 (28.00%)	0.59^*^	0.28*
Age at disease onset				
Mean (years)	5.10 ± 3.84	5.64 ± 3.48	0.59**	Mean difference 0.54; t=0.55; -1.43 to 2.51 (95%CI)
Age (in years) at disease onset				
≤1 year (n=4)	4 (12.5%)	0 (0.00%)	0.19^#^	0.12^#^
1-10 years (n=43)	22 (68.75%)	21 (84.00%)	0.31^*^	1.03^*^
≥10 years (n=10)	6 (18.75%)	4 (16.00%)	0.92^#^	1^#^
Complications				
DVT	4 (12.5%)	0 (0%)	0.19^#^	0.12^#^
Infections present	23 (71.9%)	9 (36%)	0.01^*^	5.95^*^
Evidence of steroid toxicity	21 (65.62%)	9 (36%)	0.05^*^	3.82^*^
Ascites/pleural effusion	31 (96.87%)	15 (60%)	0.002^#^	0.0006^#^
Pulmonary edema	3 (9.37%)	0 (0%)	0.33^#^	0.24^#^
Hypertension	19 (59.37%)	7 (28%)	0.04^*^	4.38^*^
Shock	5 (15.62%)	1 (4%)	0.32^#^	0.21^#^
Clinical types of disease				
SSNS	22 (68.75%)	25 (100.00%)	0.006^#^	0.0016^#^
SDNS	9 (28.12%)	6 (24%)	1.0^*^	0.12^*^
FRNS	10 (31.25%)	7 (28%)	1.0^*^	0.07^*^
IFRNS	5 (15.62%)	13 (52%)	0.008^*^	6.99^*^
SRNS	10 (31.25%)	0 (0.00%)	0.006^#^	0.0016^#^
Biochemical parameters (baseline)				
Serum creatinine (mg/dl)	2.13 ± 2.56	0.45 ± 0.13	0.002**	Mean difference 0.51; t=3.27; 0.65 to 2.70 (95%CI)
	eGFR (mL/min)	46.19 ± 31.43	115.08 ± 43.58	<0.001	Mean difference 68.89; t=7.01; -49.23 to 88.56 (95%CI)
Serum albumin(g/dl)		1.54 ± 0.46	1.96 ± 0.91	0.03	Mean difference 0.42; t=2.55; -0.04 to 0.78 (95%CI)
	Raised CRP/procalcitonin	17 (53.12%)	5 (20%)	0.02^*^	5.18^*^
High urine osmolality	9 (28.12%)	1 (4%)	0.004^#^	0.03^#^
Risk factors				
Infections	23 (71.87%)	9 (36%)	0.01^*^	5.95^*^
Hydration status (clinically)	9 (28.12%)	9 (36%)	0.73^*^	0.12^*^
Inadequate water intake	27 (84.37%)	12 (48%)	0.008^*^	6.99^*^
Hypo-albuminemia	30 (93.75%)	21 (84%)	0.45^#^	0.39^#^
Nephrotoxic drugs	28 (87.5%)	14 (56%)	0.02^*^	5.65^*^
Drug used				
Tacrolimus	9 (28.12%)	0 (0%)	0.01^#^	0.003^#^
Enalapril	25 (78.12%)	11 (44%)	0.02^*^	8.56^*^
Cyclophosphamide	1 (3.12%)	1 (4%)	0.58^#^	1^#^
Cyclosporine	3 (9.37%)	2 (8%)	0.78^#^	1^#^
Lasix/other diuretics	32 (100%)	25 (100%)	-	As all children were given diuretics, hence no different groups
Hypovolemia	13 (40.62%)	12 (48%)	0.78^*^	0.08^*^
Inotrope use	5 (15.62%)	1 (4%)	0.32^#^	0.21^#^
Antibiotic use	6 (18.75%)	5 (20%)	0.82^#^	1^#^

The common infections observed were UTI (52.63%), followed by gastroenteritis (28.07%), peritonitis (17.54%), and cellulitis (17.54%). Two out of five children with shock and AKI had septic shock requiring inotropes. A presumptive diagnosis of UTI was made based on routine urine microscopy, and urine samples were cultured in those children for a definitive diagnosis of UTI. Urine culture growth was seen in 19 samples (65.51%). The most common isolates were *Escherichia coli* (63%), followed by *Enterococcus faecalis* (16%), *Klebsiella pneumoniae* (16%), and *Acinetobacter baumannii* (5%). Blood culture was suggestive of infection in five patients. The common organisms isolated were *Escherichia coli* and *Streptococcus pneumoniae* (33% each), with others being *Staphylococcus epidermidis* and *Enterococcus faecalis* (20% each). It was observed that pre-renal/functional AKI was present in 53.13% (17 of 32), followed by renal AKI in 46.88%. Stage 2 and stage 3 AKI were more commonly observed than stage 1 (Table 2).

**Table 2:** Stage wise distribution of AKI AKI: acute kidney injury

Parameter	Frequency (n=32)	Percentage
Type of AKI		
Pre-renal	17	53.13%
Renal	15	46.88%
Stage-wise distribution of AKI		
Stage 1	8	25%
Stage 2	12	37.5%
Stage 3	12	37.5%

Out of 29 children, biopsy was done in only 10 patients due to financial constraints and refusal of consent. Of them, six (60%) were found to have focal segmental glomerulosclerosis, whereas four (40%) had minimal change disease. It was observed that six children in the sampled population had sickle cell trait (HbAS). All six of them developed AKI.

The most common presenting complaints were edema (94.74%) and decreased urine output (80.7%), followed by hypertension (54.38%). Hematuria was present in only 2.4% of children.

The indicator of the hydration status was assessed by parameters like clinical evaluation of hydration status, hemoconcentration, urine osmolality, urine potassium (K) index, and fractional excretion of sodium (FeNa). On bivariate analysis, a statistically significant difference between both groups was found in high urine osmolality (p=0.04), a urine K-index of >0.6 (p=0.007), and FeNa <0.5% (p=0.008) (Table 3).

**Table 3:** Association between hydration status and AKI (N=57) * Chi-square test, # Fisher's exact test, degree of freedom for Chi-square = 1, n = frequency in number, value in bracket () = percentage AKI: acute kidney injury, FeNa: fractional excretion of sodium (Na)

Parameters	AKI		p-value	Tests of significance values
	Present (n=32)	Absent (n=25)		
Hydration status (clinical)				
Dehydrated	9 (28.13%)	9 (36.00%)	0.73*	0.12^*^
Normal	19 (59.38)	16 (64)		
Hemo-concentration				
Present	5 (15.63%)	1 (4.00%)	0.32^#^	0.21^#^
High urine osmolality				
>800 mOsm/kg	9 (28.13%)	1 (4.00%)	0.04^#^	0.03^#^
Urine K ^+^				7.23^*^
>0.6	8 (25.00)	16 (64.00%)	0.007^*^	
FeNa				6.91^*^
<0.5	12 (37.5%)	19 (76.00%)	0.008^*^	

On multivariate analysis, low eGFR at admission, hypertension, nephrotoxic drug exposure, tacrolimus exposure, enalapril exposure, high urine potassium index, SRNS, sickle cell trait, and significant renal lesions were found to have higher odds of causing AKI (OR >1). Similarly, factors like adequacy of water intake, FeNa, IFRNS, and infections had lower odds of causing AKI (OR <1) (Table 4).

**Table 4:** Multivariate analysis of risk factors of AKI IFRNS: infrequently relapsing nephrotic syndrome, SRNS: steroid-sensitive nephrotic syndrome, AKI: acute kidney injury, eGFR: estimated glomerular filtration rate, FeNa: fractional excretion of sodium (Na)

Variable	p-value	OR	95%CI	
			Lower	Upper
eGFR	<0.0001	24.8571	5.6876	108.6
Hypertension	0.006	5.95	1.66	21.29
Nephrotoxic drug intake	0.012	5.50	1.48	20.42
Tacrolimus exposure	0.041	20.61	1.13	374.16
Enalapril exposure	0.010	4.54	1.44	14.38
Inadequate water intake	0.005	0.17	0.04	0.58
High urine K index	0.041	9.39	1.10	80.11
FeNa	0.004	0.09	0.01	0.48
IFRNS	0.005	0.17	0.04	0.58
SRNS	0.041	20.61	1.13	374.16
Infections	0.008	0.22	0.07	0.67
Sickle cell trait	0.091	12.50	0.67	233.67
Significant renal lesion	0.001	8.09	2.44	26.82

## Discussion

The incidence of AKI in NS is increasing worldwide [5,11], which is also reflected in our study (56.14%); however, some studies conducted in India have published a lower incidence of AKI [4,12]. The Chhattisgarh region falls in the CKD of unknown origin (CKDu) belt. The case series has reported adult cases of CKD without any known etiology [13]. Since pediatric data about CKDu in Chhattisgarh is lacking, a higher incidence of AKI might be caused in the initial stage of pathogenesis by the factors responsible for causing CKDu, which need to be elicited. The number of patients with pre-renal and renal AKI was observed as 17 (53.13%) and 15 (46.88%), respectively, which is higher than the study from eastern India, where the author had reported only 5% of cases of pre-renal AKI [14]. Higher environmental temperatures and lower literacy amongst parents in central India might have contributed to children's improper hydration status. Stage 2 and 3 AKI were observed more in comparison to stage 1, similar to the study by Kushwah et al. [4].

There was no statistically significant difference in patient gender between the AKI and non-AKI groups, which is consistent with prior studies [12,14-15]. According to a 10-year follow-up research study conducted in Turkey to assess complications in childhood NS, the female gender was found to be a significant risk factor for the development of AKI [16], while a prospective observational study from eastern India found the male gender as a risk factor for AKI [17].

The mean age of patients developing AKI at admission was (6.12 ± 3.90) years, while those without AKI were (7.96 ± 3.32), which was statistically significant; however, no significant association of age of onset with AKI was noted. Kim et al. and Yang et al. [15,18], however, noted that older children were at higher risk of developing AKI.

The predominant presenting complaint was edema (94.74%), followed by decreased urine output (80.7%). The IFRNS subtype had a lower risk of AKI (OR <1) and was statistically significant (p=0.008). In contrast, the steroid-dependent nephrotic syndrome and frequently relapsing nephrotic syndrome groups had an equal risk of AKI. All children in the study group with SRNS had AKI at least once during the study period (p = 0.006), which reflects studies from other parts of the country [12,14].

The most common complications in the present study were pleural effusion and ascites in 46 (80.7%), followed by infections in 32 (56.14%). Twenty-six (45.61%) had hypertension at the time of admission, with the majority being stage 1 hypertension (38.46%), followed by stage 2 (30.77%). Hypertension was associated with AKI in 19 (59.37%) cases in comparison to non-AKI (p=0.04), similar to the observations from other parts of India [14,19].

The presence of infections in children with AKI in our study (71.9%) was statistically significant (p0.01) [14]. Similar to our research, the majority of these studies have been conducted in tertiary care settings, where referral cases of infections with complications are frequent. In our study, two children out of five with shock who subsequently developed AKI had septic shock requiring inotropic support. Sepsis causes AKI by several mechanisms, like widespread inflammation, organ hypoperfusion, and the use of antibiotics and inotropes that can be nephrotoxic [20]. As a result, the conclusions drawn in the case of infections need to be done cautiously.

Blood chemistry, like higher serum creatinine and urine osmolality and lower serum albumin value and lower eGFR at admission, were analyzed between the two groups, and the above-mentioned baseline values noted in both groups were found to have a strong association with the risk factor and the causal association in AKI (p<0.05), similar to the reported prevalence [4].

Dehydration is an acute state where water loss leads to TBW deficit. Underhydration is a chronic state of low water intake where the TBW is normal due to increased serum vasopressin, which maintains water homeostasis. In underhydration, clinically, the person appears well hydrated with normal TBW but has raised urine osmolality [21]. Dehydration is a known risk factor for the development of AKI. Although many studies have proved that dehydration is a risk factor for AKI [15,22], no studies have been done in humans to establish an association between underhydration and AKI. Studies on underhydration that can cause both cellular and interstitial volume depletion, which is not clinically evident and responsible for the development of AKI in NS, need to be explored. Clinical assessment of hydration revealed 18 (31.6%) children with dehydration, four (7%) with fluid overload, and 35 (61.4%) children with normal hydration. Water intake was not adequate in the majority of the patients enrolled in the study (68.4%). Parameters like high urine osmolality, urine K+, and FeNa <0.5%, which suggest high aldosterone levels, had a significant association with AKI (p<0.01). A study done on adolescent rats by restricting water intake and hydrating them with fructose-containing beverages from infancy to look for AKI found that there was a significant fall in creatinine clearance and tubular damage as seen by increased NGAL and KIM-1 levels in the adolescent period [23]. We aimed to study underhydration, as children with NS already have a contracted intravascular compartment, and persistent underhydration would further deplete it, so hemoconcentration, urine osmolality, urine K+ index, and FeNa were taken as laboratory parameters to evaluate it. The present study fails to demonstrate a significant association between hemoconcentration AKI in NS; however, high urine osmolality, raised urine K+ index, and FeNa <0.5 suggestive of raised aldosterone levels had a significant association with AKI.

SCD is known to have renal impairment, but this is seen more in SCD as compared to sickle cell trait, and that too in adulthood [24]. But irrespective of age, AKI occurs in 4-10% of hospitalized patients with SCD and is more frequent in patients with acute chest syndrome (13.6%) than in patients with painful crisis. Factors that predispose to AKI include volume depletion, rhabdomyolysis, infections, and the use of nonsteroidal analgesics [25]. Compared with people with a normal hemoglobin phenotype, sickle cell trait was associated with a higher risk for sustained AKI [26]. Six subjects were found to have sickle cell trait (HbAS) in our study; all had AKI. They had no other symptoms pertaining to sickle cell crisis. Such a small sample is not adequate to comment upon the risk of sickle cell trait in causing AKI in NS and needs further studies.

Known factors of AKI in NS were studied, and low eGFR at admission, hypertension, nephrotoxic drug exposure, tacrolimus exposure, enalapril exposure, SRNS, and significant lesions (determined by clinical features) were found to have higher odds of causing AKI [4,11].

Limitations

As AKI has a multifactorial origin with different patient profiles and treatment protocols being followed across centers, a multicentric study with a larger sample size can help generalize results. Also, using the upcoming artificial intelligence technology to design prediction models based on data from centers worldwide can also help to get more insight [27]. Delay in presentation to us after seeking healthcare elsewhere might have led to the cohort of complicated cases and, hence, a higher incidence of AKI in our study.

## Conclusions

The present study establishes the known risk factors for AKI in children with NS along with the possible relation between underhydration and sickle cell trait, which needs further research. This underscores the importance of proper counseling for parents to maintain hydration status and screening for AKI in children on follow-up.
